# Improvements in antimicrobial reporting in Australian and New Zealand laboratories after publication of national guidelines: a follow-up survey from the Royal College of Pathologists of Australasia Quality Assurance Programs

**DOI:** 10.1093/jacamr/dlag069

**Published:** 2026-05-11

**Authors:** Juliet Elvy, Katherine Ryan, Melanie Otormin, Menuk Jayawardena, Maryza Graham

**Affiliations:** Royal College of Pathologists of Australasia Quality Assurance Programs (RCPAQAP), Microbiology Discipline, Saint Leonards, NSW, Australia; Department of Microbiology, Awanui Labs, Dunedin Hospital, Great King Street, Dunedin 9016, New Zealand; Royal College of Pathologists of Australasia Quality Assurance Programs (RCPAQAP), Microbiology Discipline, Saint Leonards, NSW, Australia; Royal College of Pathologists of Australasia Quality Assurance Programs (RCPAQAP), Microbiology Discipline, Saint Leonards, NSW, Australia; Royal College of Pathologists of Australasia Quality Assurance Programs (RCPAQAP), Microbiology Discipline, Saint Leonards, NSW, Australia; Department of Microbiology, Monash Pathology, Clayton, VIC, Australia; Victorian Infectious Diseases Reference Laboratory, The Peter Doherty Institute of Infection and Immunity, Melbourne, VIC, Australia; Department of Infectious Diseases, University of Melbourne at the Peter Doherty Institute for Infection and Immunity, Melbourne, VIC, Australia

## Abstract

**Objectives:**

In 2017, a Royal College of Pathologists of Australasia Quality Assurance Programs (RCPAQAP) laboratory survey demonstrated suboptimal antimicrobial susceptibility reporting practices. In response, the RCPA published Selective Reporting of Antimicrobials Guidelines to support selective and cascade reporting of antimicrobials. We sought to revisit laboratory antimicrobial reporting practices since these guidelines were published.

**Materials and methods:**

We carried out a repeat of the laboratory survey in 2023 and compared results to the previous 2017 report. We also studied antimicrobial reporting practices from two routine external quality assurance (EQA) samples dispatched by the RCPAQAP.

**Results:**

In 2023, compared with 2017, there was an overall improvement in selective reporting and a reduction in reporting of antibiotics inappropriate for bacteraemia. For a fully susceptible *Escherichia coli* in blood cultures, fewer laboratories over-reported antibiotics [29/62 (47%) and 55/84 (65%), respectively, *P* = 0.03] or reported ciprofloxacin [13/55 (24%) and 41/73 (56%), respectively, *P* = 0.03]. Similarly, for a fully susceptible *E. coli* in urine, fewer laboratories reported amoxicillin-clavulanate [15/57 (26%) and 44/90 (49%), respectively, *P* = 0.006] and ciprofloxacin [6/51 (12%) and 12/57 (21%), respectively, *P* = 0.21]. For a wild-type *Klebsiella pneumoniae* in blood culture, fewer laboratories reported inappropriate antibiotics for bacteraemia [11/94 (12%) and 1/62 (2%), *P* = 0.02].

**Conclusions:**

We demonstrate an improvement in selective antimicrobial reporting following the publication of national antimicrobial reporting guidelines for laboratories, but opportunities for further improvement and standardization remain. This work is encouraging and highlights how microbiology laboratories can be supported to contribute to antimicrobial stewardship efforts.

## Introduction

Selective reporting of antimicrobial susceptibilities intentionally limits which susceptible antimicrobials are reported to the clinician, such as those with an unnecessary broad spectrum of activity. Published literature shows that selective reporting can influence prescribing practice, reduce antimicrobial usage and decrease antimicrobial resistance rates,^1–7^ and the practice is widely recommended.^8–14^

A 2017 Royal College of Pathologists of Australasia Quality Assurance Programmes (RCPAQAP) survey of Australian and Aotearoa New Zealand (NZ) laboratories identified deficiencies in selective reporting. Ninety-four laboratories responded to this survey in 2017, with 65% (55/84) of laboratories over-reporting at least one antimicrobial for a fully susceptible *Escherichia coli* in blood culture and 12% (10/84) reporting antimicrobials inappropriate for bacteraemia.^15^ In response, the Royal College of Pathologists of Australasia (RCPA) published the 2019 RCPA Selective Reporting of Antimicrobials Guidelines,^16,17^ later adapted and published by the New Zealand National Antimicrobial Susceptibility Testing Committee (NZNAC) for the NZ context.^18,19^ At the time, these were the first national selective reporting implementation guidelines of this type in the world.^16,20^

In view of the importance of selective reporting to support antimicrobial stewardship (AMS), we sought to revisit practices since the publication of the guidelines. We did this by repeating the 2017 survey in 2023, and also by interrogating antimicrobial reporting practices from two routine external quality assurance (EQA) samples dispatched by the RCPAQAP.

## Materials and methods

### Part 1: Antimicrobial Reporting Questionnaire

In 2023, Australian and NZ laboratories enrolled in the RCPAQAP bacteriology EQA were invited to respond to an electronic questionnaire. Questions were as previously published.^1^ Briefly, laboratories were asked to indicate which antibiotics, from a provided list, were reported for a fully susceptible *E. coli* in blood cultures, a fully susceptible *E. coli* in urine and a *Klebsiella pneumoniae* only resistant to ampicillin/amoxicillin in blood cultures. Laboratories were also asked to indicate whether they used EUCAST, CLSI, Calibrated Dichotomous Sensitivity (CDS) breakpoints (or a combination thereof) and if selective/cascade reporting principles were followed.

Contradictory duplicate entries for a given laboratory were removed while concordant duplicate responses were consolidated into a single response. The two-proportion *z*-test or Fisher’s exact test was used to calculate statistical significance for differences in the proportion of laboratories reporting an antibiotic in 2017 compared with 2023.

### Definitions

Definitions of over-reporting and inappropriate reporting were as stated previously, although norfloxacin was not included as a reporting option in the 2023 questionnaire.^1^

Inappropriate reporting was defined as reporting of antimicrobials not appropriate for the site of infection. For isolates in blood cultures we defined this as reporting of cefalexin, nitrofurantoin or trimethoprim as these antimicrobials do not generally achieve sufficient blood levels to adequately treat bacteraemia.^13^

Over-reporting was defined as reporting of broad-spectrum antimicrobials on isolates susceptible to narrow-spectrum antimicrobials. We defined ‘ideal’ reporting for a fully susceptible *E. coli* in blood cultures as reporting of ampicillin/amoxicillin, cefazolin/ceftriaxone/cefuroxime, gentamicin and co-trimoxazole with additional reporting of amoxicillin/clavulanate or ampicillin/sulbactam if the blood culture isolate was *K. pneumoniae*. For a fully susceptible *E. coli* in urine we considered reporting of ampicillin/amoxicillin, cefalexin, gentamicin, nitrofurantoin and trimethoprim as ideal. Over-reporting was defined as reporting any antibiotic not considered ‘ideal’.

### Part 2: RCPAQAP EQA dispatch

The RCPAQAP EQA electronic results portal requires laboratories to enter antimicrobial susceptibility results as well as indicate which are reported to the clinician. We collated routine reporting practices for two EQA isolates dispatched in 2023 (a fully susceptible *E. coli* in urine) and 2025 (amoxicillin/ampicillin resistant *K. pneumoniae* in blood culture) to determine the proportion of participants testing and reporting to the clinician a range of antimicrobials.

## Results

### Part 1: Antimicrobial Reporting Questionnaire

The electronic questionnaire was distributed to 156 RCPAQAP enrolled laboratories, with an overall response rate of 40% (62/156). Fifty (81%) responses were from Australia and 12 (19%) were from NZ.

For all scenarios combined, fewer laboratories over-reported at least one antibiotic in 2023 compared with 2017 [73% (45/62) and 82% (77/94), respectively, *P* = 0.25]. Similarly, for a fully susceptible *E. coli* in blood cultures, fewer laboratories over-reported antibiotics in 2023 compared with 2017 [47% (29/62) and 65% (55/84) respectively, *P* = 0.03] and fewer laboratories performing testing for amoxicillin-clavulanate, ciprofloxacin, piperacillin-tazobactam and meropenem reported these results (Table 1).

**Table 1.** Comparison of laboratories reporting antimicrobials in 2017 and 2023

Scenario	Antibiotic	Number (%) of laboratories testing and reporting the antibiotic		*P* value (comparing 2017 with 2023)
		2017	2023	
Fully susceptible *E. coli* in blood culture	Amoxicillin/ampicillin	82/82 (100%)	58/59 (98%)	0.32
	Amoxicillin/clavulanate	31/79 (39%)	16/58 (28%)	0.18
	Ceftriaxone	41/73 (56%)	28/54 (52%)	0.67
	Ciprofloxacin	33/79 (42%)	13/55 (24%)	0.03
	Co-trimoxazole	41/69 (59%)	36/54 (67%)	0.34
	Piperacillin/tazobactam	24/69 (35%)	11/54 (20%)	0.07
	Meropenem	10/65 (15%)	5/56 (9%)	0.44
	Nitrofurantoin	0	0	n/a
	Trimethoprim	2/41 (5%)	1/29 (3%)	1.0
	Over-reported antibiotics	55/84 (65%)	29/62 (47%)	0.03
	Inappropriate reporting	6/94 (6%)	3/62 (5%)	0.68
Fully susceptible *E. coli* in urine	Amoxicillin/ampicillin	92/93 (99%)	57/58 (98%)	1.0
	Amoxicillin/clavulanate	44/90 (49%)	15/57 (26%)	0.006
	Trimethoprim	91/92 (99%)	55/57 (96%)	0.31
	Nitrofurantoin	80/87 (92%)	53/55 (96%)	0.41
	Cefalexin	64/65 (98%)	44/45 (98%)	1.0
	Ciprofloxacin	12/57 (21%)	6/51 (12%)	0.21
	Co-trimoxazole	16/37 (43%)	7/29 (24%)	0.14
	Piperacillin/tazobactam	1/32 (3%)	1/59 (2%)	1.0
	Meropenem	1/37 (3%)	0/34	1.0
	Over-reported antibiotics	59/94 (63%)	32/62 (52%)	0.15
*K. pneumoniae* in blood culture, resistant to only ampicillin/amoxicillin	Amoxicillin/ampicillin	80/81 (99%)	56/58 (97%)	0.56
	Amoxicillin/clavulanate	63/80 (79%)	51/57 (89%)	0.14
	Ceftriaxone	42/73 (58%)	33/55 (60%)	0.83
	Ciprofloxacin	35/81 (43%)	17/56 (30%)	0.10
	Co-trimoxazole	41/68 (60%)	36/53 (68%)	0.34
	Piperacillin/tazobactam	28/71 (39%)	16/52 (31%)	0.34
	Meropenem	9/65 (14%)	3/58 (5%)	0.13
	Nitrofurantoin	2/30 (7%)	0/25 (0%)	0.50
	Trimethoprim	6/39 (15%)	1/29 (3%)	0.21
	Over-reported antibiotics	54/94 (57%)	28/62 (45%)	0.14
	Inappropriately reported antibiotics	11/94 (12%)	1/62 (2%)	0.02

For a fully susceptible *E. coli* in urine, fewer laboratories over-reported antibiotics in 2023 compared with 2017 [52% (32/62) and 63% (59/94) respectively, *P* = 0.15], and reporting rates for amoxicillin-clavulanate, ciprofloxacin and co-trimoxazole also decreased (Table 1).

Similarly, for the *K. pneumoniae* in blood culture, fewer laboratories unnecessarily reported broad-spectrum antibiotics such as ciprofloxacin, piperacillin-tazobactam and meropenem, while more laboratories reported amoxicillin-clavulanate and co-trimoxazole (Table 1).

Overall, 3/62 (5%) laboratories reported one or more antimicrobials considered inappropriate for the treatment of bacteraemia in 2023, namely cefalexin and trimethoprim, compared with 10/84 (12%) in 2017, *P* = 0.24.

None of the laboratories over-reporting ciprofloxacin or meropenem, and only one laboratory over-reporting piperacillin-tazobactam, were from NZ.

For a full list of submitted responses for each antibiotic, see Supplementary Table S1 (available as Supplementary data at *JAC-AMR* Online).

#### Susceptibility testing method

In 2023, 26% (16/62) of responding laboratories used CLSI breakpoints, 44% (27/62) used EUCAST and 8% (5/62) used CDS; the remaining 22% (14/62) used a hybrid of these methods. This reflects a shift from CLSI towards EUCAST, and a decline in the use of CDS across Australian laboratories, since 2017. Only one laboratory (2%) indicated that selective/cascade reporting was not in use, compared with 8/94 (9%) in 2017 (*P* = 0.08), and this same laboratory over-reported meropenem for two of the three scenarios.

### Part 2: RCPAQAP microbiology external quality assurance (EQA) surveys

In 2023, 136 Australian and NZ laboratories participated in an RCPAQAP urine dispatch which included a fully susceptible *E. coli* (117 Australian, 19 NZ). Eleven laboratories (11/31, 35%) indicated they would test, and report, ciprofloxacin compared with 122/125 (98%), 124/128 (97%) and 103/110 (94%) for amoxicillin, trimethoprim and nitrofurantoin, respectively (table 2). Reporting for the EQA urine dispatch was similar to reporting in the questionnaire with two exceptions (where reporting rates were reported to be lower in the questionnaire than for the EQA): amoxicillin-clavulanate and gentamicin. Only one laboratory tested and reported meropenem.

**Table 2.** Antibiotics tested and reported in RCPAQAP EQA dispatches compared to the responses to the 2023 questionnaire

Antibiotic	Fully susceptible *E. coli* in urine				*K. pneumoniae* in blood culture only resistant to ampicillin/amoxicillin			
	RCPAQAP 2023 urine EQA dispatch		RCPAQAP 2023 questionnaire		RCPAQAP 2025 blood culture EQA dispatch		RCPAQAP 2023 questionnaire	
	No. (%) tested	Tested and reported (%)	Tested and reported (%)	*P* value	No. (%) tested	Tested and reported (%)	Tested and reported (%)	*P* value
Ampicillin/amoxicillin	125/136 (92%)	122/125 (98%)	57/58 (98%)	0.816	96/100 (96%)	95/96 (99%)	56/58 (97%)	0.37
Amoxicillin-clavulanate	91/136 (67%)	56/91 (62%)	15/57 (26%)	<0.001	87/100 (87%)	82/87 (94%)	51/56 (91%)	0.48
Cefalexin	94/136 (69%)	91/94 (97%)	44/45 (98%)	0.81	1/100 (1%)	1/1 (100%)	1/10 (2%)	n/a
Ceftriaxone	—	—	—	—	69/100 (69%)	60/69 (87%)	33/55 (60%)	<0.001
Ciprofloxacin	31/136 (23%)	11/31 (35%)	6/51 (12%)	0.012	63/100 (63%)	50/63 (79%)	17/56 (30%)	<0.001
Fosfomycin	1/136 (0.7%)	0/1	0/59	N/A	—	—	—	—
Gentamicin	86/136 (63%)	80/86 (93%)	34/59 (58%)	<0.001	93/100 (93%)	84/93 (90%)	50/56 (89%)	0.84
Meropenem	13/136 (10%)	1/13 (8%)	0/34	0.29	38/100 (38%)	18/38 (47%)	3/58 (5%)	<0.001
Nitrofurantoin	110/136 (81%)	103/110 (94%)	53/55 (96%)	0.55	3/100 (3%)	0/3	0/25	N/A
Piperacillin-tazobactam	9/136 (6%)	1/9 (11%)	1/59 (2%)	0.22	44/100 (44%)	29/44 (66%)	16/52 (31%)	<0.001
Trimethoprim	128/136 (94%)	124/128 (97%)	55/57 (96%)	0.9	6/100 (6%)	2/6 (33%)	1/29 (3%)	n/a
Over-reported antibiotics	—	70/136 (51%)	32/62 (52%)	0.99	—	62/100 (62%)	28/62 45%	0.036
Inappropriately reported antibiotics	—	N/A	N/A		—	2/100 (2%)	1/62 (2%)	0.85

For the 2025 RCPAQAP blood culture dispatch, which included a *K. pneumoniae* isolate resistant only to amoxicillin, 100 laboratories participated. Sixty-two laboratories (62/100, 62%) over-reported antibiotics in the EQA dispatch compared with 28/62 laboratories (45%, *P* = 0.036) in the survey. Fifty laboratories (50/63, 79%) indicated they would test and report ciprofloxacin compared with 82/87 (94%) reporting amoxicillin-clavulanate and 84/93 (90%) reporting gentamicin. Eighteen laboratories (18/38, 47%) over-reported meropenem, with three inappropriately reporting cefalexin or trimethoprim. Testing and reporting for the EQA dispatch differed to the questionnaire for ceftriaxone, ciprofloxacin, piperacillin-tazobactam and meropenem, all with more laboratories reporting in the EQA dispatch than in the questionnaire. However, the number of laboratories reporting these four antimicrobials were lower when considered as a proportion of the total number of laboratories enrolled in the EQA dispatch overall (60/100, 50/100, 29/100 and 18/100, respectively).

## Discussion

We have demonstrated that most Australian and NZ laboratories are reporting antimicrobials in line with the RCPA and NZNAC selective reporting guidelines,^17,19^ with consistently fewer laboratories over-reporting broad-spectrum antimicrobials for susceptible organisms and fewer laboratories reporting antibiotics inappropriate for bacteraemia. In particular, unnecessary reporting of meropenem, ciprofloxacin, piperacillin-tazobactam and amoxicillin-clavulanate have decreased, while reporting of narrower spectrum agents has stayed the same.

The importance of selective reporting of antimicrobial susceptibilities is widely accepted, but there are few national antibiotic reporting guidelines and studies measuring their impact are limited.^20^ Despite the many barriers that prevent adoption of consensus reporting guidelines, we show consistent improvement in reporting practices since 2017 and since the RCPA and NZNAC guidelines were published.^17,19,20^

The questionnaire relies on the self-reported practices by laboratory personnel participating in our EQA programmes. Given the inherent limitations of this methodology, we included actual reporting practices as determined through two routine EQA surveys dispatched in 2023 and 2025. It is reassuring to confirm from the EQA data that for a fully susceptible *E. coli* in urine only a minority of laboratories tested and reported ciprofloxacin, meropenem, piperacillin-tazobactam and fosfomycin (Table 2). By contrast, as expected, most laboratories that tested trimethoprim and nitrofurantoin reported these commonly recommended narrow-spectrum urinary antibiotics. Overall, reporting practices were similar between the EQA and the questionnaire responses. However, for the fully susceptible *E. coli* in urine fewer laboratories reported amoxicillin-clavulanate in our questionnaire compared with the EQA (Table 2). Similarly for the *K. pneumoniae* in blood culture, fewer laboratories reported ciprofloxacin and meropenem in the questionnaire responses than for the EQA. This could be because despite being aware of the desired or ‘correct’ response for the questionnaire, laboratories continue to over-report in practice.

Another limitation of our study is the lower response rate (40%) compared with the previous 2017 survey (60%). Although we have not explored the reasons for this, it is likely that increasing laboratory workloads and staffing pressures have contributed. While we tried to keep the survey short, completion still took time and was entirely voluntary to carry out.

Finally, we did not confirm to what degree the improvements in reporting practices were directly due to the published RCPA/NZNAC guidelines rather than increasing general awareness and acceptability of selective reporting over time. However, the publication of the guidelines was accompanied by intentional wide-reaching national dissemination and education of RCPA fellows, clinical microbiologists and laboratory staff through written information and presentations,^20^ which will probably have contributed to the improvements seen.

This study provides encouraging evidence that Australian and NZ laboratories are increasingly adopting selective antimicrobial reporting that can support AMS efforts. We demonstrate an improvement in selective antimicrobial reporting following the publication of national antimicrobial reporting guidelines but also highlight the opportunities for further improvement and standardization.

## Supplementary Material

dlag069_Supplementary_Data

## Supplementary data

Table S1 is available as Supplementary data at *JAC-AMR* Online.
