# Supplementary material for: Improvements in antimicrobial reporting in Australian and New Zealand laboratories after publication of national guidelines: a follow-up survey from the Royal College of Pathologists of Australasia Quality Assurance Programs
Source: JAC Antimicrob Resist. 2026 May 11;8(3):dlag069. doi: 10.1093/jacamr/dlag069 (PMC13158014; doi:10.1093/jacamr/dlag069)
Supplement: dlag069_Supplementary_Data [file dlag069_supplementary_data.docx]

Supplementary table S1. Full list of responses for each antibiotic against the respective scenarios. Bracketed results indicate responses only from NZ laboratories.

|  | Fully susceptible *E. coli* in blood culture | | | | Fully susceptible *E. coli* in urine | | | | *K. pneumoniae* only resistant to ampicillin/amoxicillin in blood culture | | | |
| --- | --- | --- | --- | --- | --- | --- | --- | --- | --- | --- | --- | --- |
| Antibiotic | Not tested | Tested but not reported | Reported | % reported | Not tested | Tested but not reported | Reported | % reported | Not tested | Tested but not reported | Reported | % reported |
| Amikacin | 16(7) | 46(5) | 0 | 0% | 32(10) | 26(2) | 1 | 2% | 18(6) | 44(6) | 0 | 0% |
| Amoxicillin /ampicillin | 3(1) | 1 | 58(11) | 94% | 1(1) | 1 | 57 (11) | 97% | 4(1) | 2 | 56(11) | 90% |
| Amoxicillin/clavulanate | 4(1) | 42(8) | 16(3) | 26% | 2 | 42(9) | 15(3) | 25% | 6(1) | 5 | 51(11) | 82% |
| Aztreonam | 45(7) | 16(5) | 1 | 2% | 55(9) | 4(3) | 0 | 0% | 46(6) | 16(6) | 0 | 0% |
| Cefalexin | 48(11) | 11(1) | 3 | 5% | 14(3) | 1 | 44(9) | 75% | 52(11) | 9(1) | 1 | 2% |
| Cefazolin | 28(12) | 18 | 16 | 26% | 43(12) | 7 | 9 | 15% | 29(11) | 20(1) | 13 | 21% |
| Cefepime | 15(5) | 46(7) | 1 | 2% | 29(9) | 30(3) | 0 | 0% | 18(5) | 43(7) | 1 | 2% |
| Cefotaxime | 45(9) | 14(3) | 3 | 5% | 49(12) | 10 | 0 | 0% | 49(11) | 9(1) | 4 | 6% |
| Cefoxitin | 24(5) | 38(7) | 0 | 0% | 36(9) | 23(3) | 0 | 0% | 30(5) | 32(7) | 0 | 0% |
| Ceftazidime | 17(4) | 45(8) | 0 | 0% | 31(8) | 28(4) | 0 | 0% | 16(4) | 45(8) | 1 | 2% |
| Ceftriaxone | 8(1) | 26(7) | 28(4) | 45% | 21(5) | 37(7) | 1 | 2% | 7(1) | 22(8) | 33(3) | 53% |
| Cefuroxime | 51(2) | 5(2) | 7(7) | 11% | 53(6) | 5(5) | 1(1) | 2% | 51(3) | 3(2) | 8(7) | 13% |
| Ciprofloxacin | 7(1) | 42(11) | 13 | 21% | 8(2) | 45(10) | 6 | 10% | 6(1) | 39(11) | 17 | 27% |
| Co-trimoxazole | 8(2) | 18(1) | 36(9) | 58% | 30(8) | 22(2) | 7(2) | 12% | 9(2) | 17(2) | 36(8) | 58% |
| Ertapenem | 40(5) | 22(7) | 0 | 0% | 54(9) | 5(3) | 0 | 0% | 41(5) | 21(7) | 0 | 0% |
| Fosfomycin | 58(11) | 4(1) | 0 | 0% | 54(11) | 5(1) | 0 | 0% | 60(12) | 2 | 0 | 0% |
| Gentamicin | 6(1) | 6(3) | 50(8) | 81% | 17(5) | 8(2) | 34(5) | 58% | 6(1) | 6(3) | 50(8) | 81% |
| Meropenem | 6(2) | 51(10) | 5 | 8% | 25(5) | 34(7) | 0 | 0% | 4(1) | 55(11) | 3 | 5% |
| Nitrofurantoin | 37(10) | 25(2) | 0 | 0% | 4 | 2 | 53(12) | 90% | 37(10) | 25(2) | 0 | 0% |
| Piperacillin/tazobactam | 8(4) | 43(8) | 11 | 18% | 29(8) | 29(4) | 1 | 2% | 10(3) | 36(8) | 16(1) | 26% |
| Ticarcillin/clavulanate | 46(12) | 16 | 0 | 0% | 49(12) | 10 | 0 | 0% | 45(12) | 17 | 0 | 0% |
| Tobramycin | 22(10) | 40(2) | 0 | 0% | 37(11) | 22(1) | 0 | 0% | 24(9) | 38(3) | 0 | 0% |
| Trimethoprim | 33(10) | 28(2) | 1 | 2% | 2 | 2 | 55(12) | 93% | 33(10) | 28(2) | 1 | 2% |
